# Impact of media supplements FGF2, LIF and IGF1 on the genome activity of porcine embryos produced in vitro

**DOI:** 10.1038/s41598-024-57865-7

**Published:** 2024-03-25

**Authors:** Alexandra Rosenbaum Bartkova, Lucie Nemcova, Frantisek Strejcek, Ahmed Gad, Veronika Kinterova, Martin Morovic, Michal Benc, Radek Prochazka, Jozef Laurincik

**Affiliations:** 1 Constantine the Philosopher University in Nitra, Nitra, Slovakia; 2 Institute of Animal Physiology and Genetics, Czech Academy of Sciences, Liběchov, Czech Republic; 3 Animal Reproduction and Biotechnology Laboratory (ARBL), Department of Biomedical Sciences, Colorado State University, Fort Collins, CO USA; 4 Department of Animal Production, Faculty of Agriculture, Cairo University, Giza, Egypt

**Keywords:** Cell biology, Developmental biology, Molecular biology

## Abstract

In this article, we focused on the impact of precisely chemically modified FLI maturation medium enriched with fibroblast growth factor 2 (FGF2), leukemia inhibitory factor (LIF), insulin-like growth factor 1 (IGF1), and polyvinyl alcohol (PVA) and its potential to improve the efficiency of in vitro production of porcine embryos. We hypothesized that enhancing the composition of the maturation medium could result in an elevated production of embryos in vitro and can affect EGA*.* FLI medium resulted in a significantly higher rate of oocyte blastocyst maturation and formation compared to the control DMEM medium. In addition, immunocytochemical labelling confirmed the detection of UBF in 4-cell FLI parthenogenic embryos, suggesting similarities with natural embryo development. Through RNAseq analysis, upregulated genes present in 4-cell FLI embryos were found to play key roles in important biological processes such as cell proliferation, cell differentiation, and transcriptional regulation. Based on our findings, we demonstrated the positive influence of FLI medium in the evaluation of in vitro embryo production, EGA detection, transcriptomic and proteomic profile, which was confirmed by the positive activation of the embryonal genome in the 4-cell stage of parthenogenetically activated embryos.

## Introduction

The production of embryos in laboratory conditions (IVP) is a challenging process for reproductive medicine and embryo technology specialists. However, it serves as a valuable research technique in fields such as embryogenesis research, genetically modified organisms generation, xenotransplantation, and transgenesis. Thus, IVP has gained significant attention in biotechnology and remains an important topic of study today as evidenced by publications^1–4^. The IVP process includes three or four technical steps—a collection of matured or immature oocytes, in vitro maturation (IVM) of immature oocytes, in vitro fertilisation (IVF) of matured oocytes, and in vitro embryo cultivation (IVC)^5^. In several species that are used as model organisms, including pigs, cattle, sheep, etc., it has been shown that the in vitro embryo developmental competence is consistently lower than in vivo^6,7^.

The success of IVP depends on numerous factors such as oocyte size and quality, its molecular determinants such as organelle localization, genes and proteins expression, manipulator skills, in vitro conditions, and composition of maturation and cultivation media. In our laboratory conditions, the porcine oocytes and embryos are used as a model system. The production of in vitro porcine embryos has become an essential tool for animal breeding and genetic research^8^. The ability to manipulate and study early embryonic development has allowed for significant advancements in the field of reproductive biology. The potential of the development of in vitro produced porcine embryos remains a topic of debate among researchers, even though it has become an essential tool for examining early embryonic development and enhancing animal breeding programs. The process involves fertilizing matured oocytes in a lab setting outside the female reproductive tract, which requires careful consideration of factors like culture media, temperature, and gas concentrations^9^. However, there exist various challenges in this process as studies have demonstrated that blastocysts developed under such conditions are less competent compared to those developed naturally in vivo^10^. Roughly 40% of presumed zygotes develop to the blastocyst stage for embryo transfer, with fewer cells observed in these embryos compared to those that progressed naturally. To ensure the developmental ability of in vitro embryos, it is imperative to examine their transcriptional profile during pre-implantation stages and identify possible factors that may affect their competence^9,11,12^.

In vitro maturation is being considered as a potential option to increase the success rate of IVP. Through IVM, a large number of matured oocytes can be sourced in a safe and resource-efficient manner for activation or fertilization in vitro. To perform successful IVM, prepubertal follicles measuring between 1 to 10 mm in diameter need to be retrieved depending on the organism's specifications according to Edwards et al.^13^. A controlled environment and optimized maturation medium are essential elements for successful IVM, which support nuclear and cytoplasmic maturation critical for embryogenesis and fertilization, as further explained by Hatirnaz et al*.* and Vuong et al.^14,15^. The developmental potential of in vitro produced porcine embryos has greatly improved due to advances in technology and techniques. Research has shown that the addition of specific growth factors to the media and the optimization of culture conditions can support the development of embryos and at the same time increase the rate of blastocyst formation^8,16^. Optimizing the individual cultivation media therefore proved to be a very important step. There has recently been a significant improvement in porcine oocyte IVM using media such as TCM-199 or DMEM, resulting in improved porcine embryo production. This advance was achieved by Yuan et al. who developed a chemically defined medium known as FLI medium. Instead of using fetal bovine serum (FBS), FLI medium contains fibroblast growth factor 2 (FGF2), leukemia inhibitory factor (LIF), insulin-like growth factor 1 (IGF1), and polyvinyl alcohol (PVA)^17^.

Enhancing developmental potential requires a detailed understanding of transcription activity during early embryonic development in porcine embryos beyond merely optimizing culture conditions. The examination of the transcriptome has showcased variations in gene expression between porcine embryos created through in vivo and in vitro methods. This indicates that there should be more investigation into detecting crucial genes responsible for embryonic development during its early stages^10^. The purpose of this research was to compare two different maturation media: Dulbecco's modified Eagle's medium (DMEM, Biowest, L0101-500) and FLI. These media differ in their composition and individual concentrations of supplementary supplements. For example, EGF is present at 50 ng/ml in DMEM but only at 10 IU/ml in FLI; IGF is present at a concentration of 100 ng/ml in DMEM compared to 20 ng/ml in FLI; FGF2 has a concentration of 5 ng/ml in DMEM, while it is 40 ng/ml in FLI; The concentration of LIF varies from none (0) for DMEM to 20 ng/ml for FLI. In the experimental part of our study, we specifically investigated how these media affected the maturation process, early embryonic growth, proteomic profile, and gene expression. In addition, we sought to assess specific parameters that influence the development potential of oocytes when cultured in vitro conditions.

## Results

### Oocyte maturation and embryonic development

Assessing experimental groups in our laboratory conditions generated oocyte maturation to the MII stage success rate of 83.60 ± 1.54 in DMEM and 92.40% ± 0.67 in FLI (Table 1). Differences in the experimental groups were evaluated by ANOVA with Tukey’s post-test and the results were statistically significant (p < 0.01).

**Table 1:** Maturation ability of porcine oocytes maturated in DMEM and FLI medium.

Type of medium	No. of oocytes	Oocyte quantity in MII stage % ± SEM
DMEM	500	83.60 ± 1.54^a^
FLI	500	92.40 ± 0.67^b^

Five replicates of this experiment were performed. In brackets, numbers of oocytes are stated. Data are expressed in percentages ± SEM.Within a column, values with different small letters (a, b) are significantly different with P < 0.01.

By evaluating the parthenogenetic development of oocytes cultivated in the DMEM and FLI media after ionomycin activation, we were able to compare embryonic development. In the DMEM group, the cleavage rate was 78.01% ± 1.83 and in the FLI group, it was 90.22% ± 0.71. The blastocyst rate was 32.54% ± 0.72 in the DMEM group and 45.76% ± 1.39 in the FLI group (Table 2). Significant differences were observed between both experimental groups (p < 0.01).

**Table 2:** Development ability of porcine embryos parthenogenetically activated from oocytes maturated in DMEM and FLI medium.

Type of medium	No. of activated oocytes	Cleavage rate % ± SEM	Blastocyst rate % ± SEM
DMEM	380	78.01 ± 1.83^a^	32.54 ± 0.72^a^
FLI	390	90.22 ± 0.71^b^	45.76 ± 1.39^b^

Five repetitions of this experiment were performed. In brackets, the numbers of oocytes are stated. Data are expressed in percentages ± SEM. Within a column, values with different small letters (a, b) are significantly different with P < 0.01.

### Protein profiling of porcine oocytes

The subject of proteomic analysis was the comparison of proteomic profiles of oocytes maturated in two different culture media, DMEM and FLI. A group of 20 oocytes from each media was used for analysis. Assays were performed in 4 replicates. In the DMEM experimental group, we identified a total of 82 proteins, in the FLI group, 53 proteins were detected, but 29 proteins were common.

Identified proteins were divided into 3 groups depending on their biological activity and molecular function described in the UniProt database. In the first group (GE), proteins involved in gene expression (DNA replication, transcription, translation, etc.) were selected. The second group of proteins (CM) consisted of cytoskeletal and microtubular proteins, as well as proteins involved in meiotic spindle formation. In the third group (ZP), zona pellucida proteins were enlisted.

**Figure 1:**
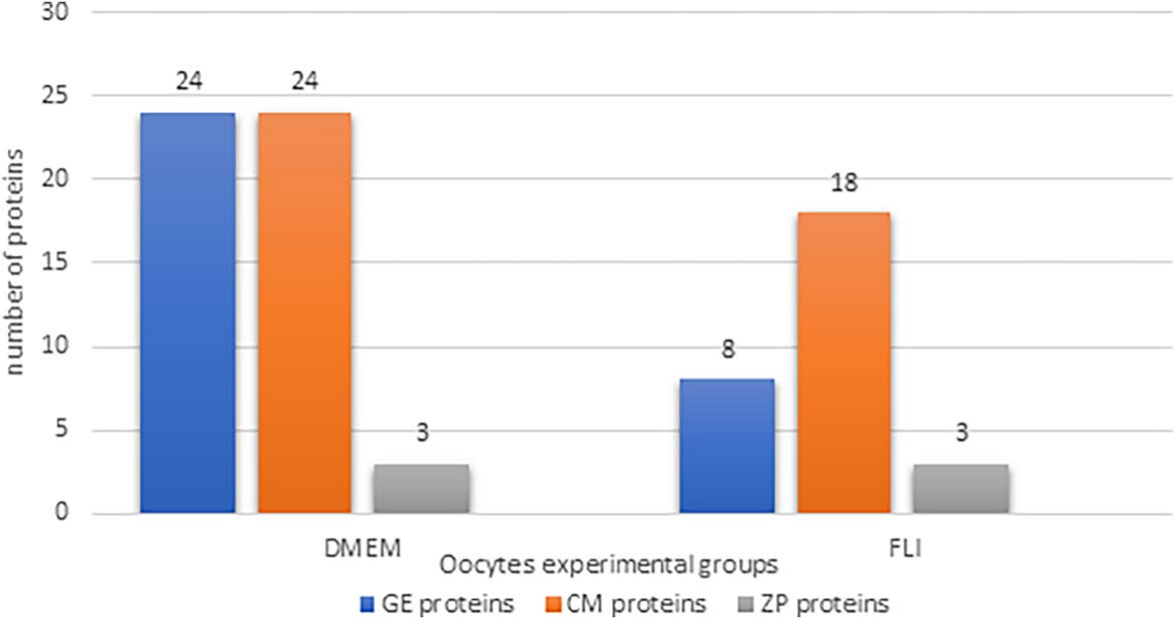
Differences in number of selected groups of proteins in DMEM and FLI oocytes identified by tandem mass spectrometry. *GE* proteins involved in gene expression (DNA replication, transcription, translation, etc.), *CM* consisted of cytoskeletal and microtubular proteins, *ZP* zona pellucida proteins.

After a comparison of individual groups of proteins between experimental groups of oocytes, significant differences in the number of proteins involved in gene expression and cytoskeletal proteins were observed. The number of GE proteins significantly decreased from 24 in DMEM to 8 in the FLI group of oocytes. In the CM group, the number of identified proteins slightly dropped from 24 in DMEM to 18 in FLI oocytes. The selected type of maturation medium did not affect the number (3) of ZP proteins in analysed oocytes (Fig. 1).

**Figure 2:**
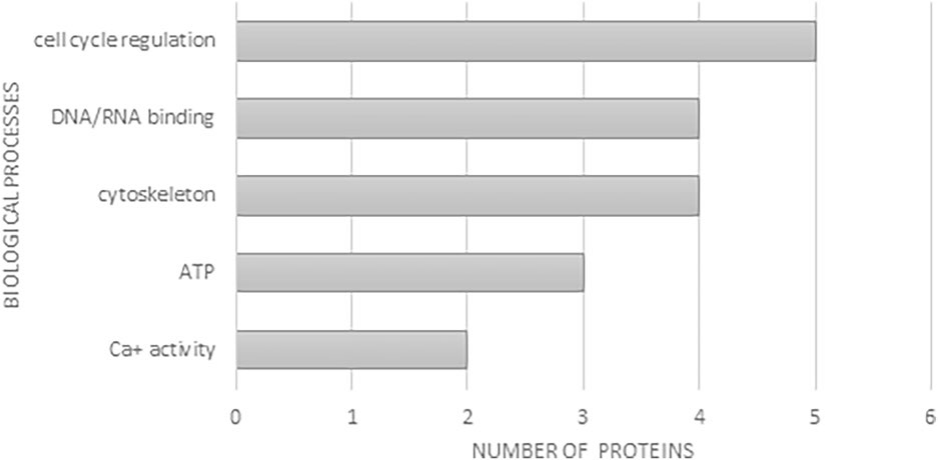
Biological processes supported by proteins detected in the FLI experimental group.

Following the design of transcriptomic profiling of 4-cell and 8-cell embryos derived from oocytes cultivated in DMEM and FLI mediums, we focused our analyses on biological processes correlated with identified proteins and we noticed that proteins in the FLI group are involved in biological processes connected with cell cycle regulation, DNA/RNA binding, cytoskeleton organisation, ATP and Ca^2+^ activity. These facts can hypothetically correlate with successful cytoplasmic maturation and higher developmental potential of oocytes maturated in the FLI medium (Fig. 2).

### Immunocytochemistry

**Figure 3:**
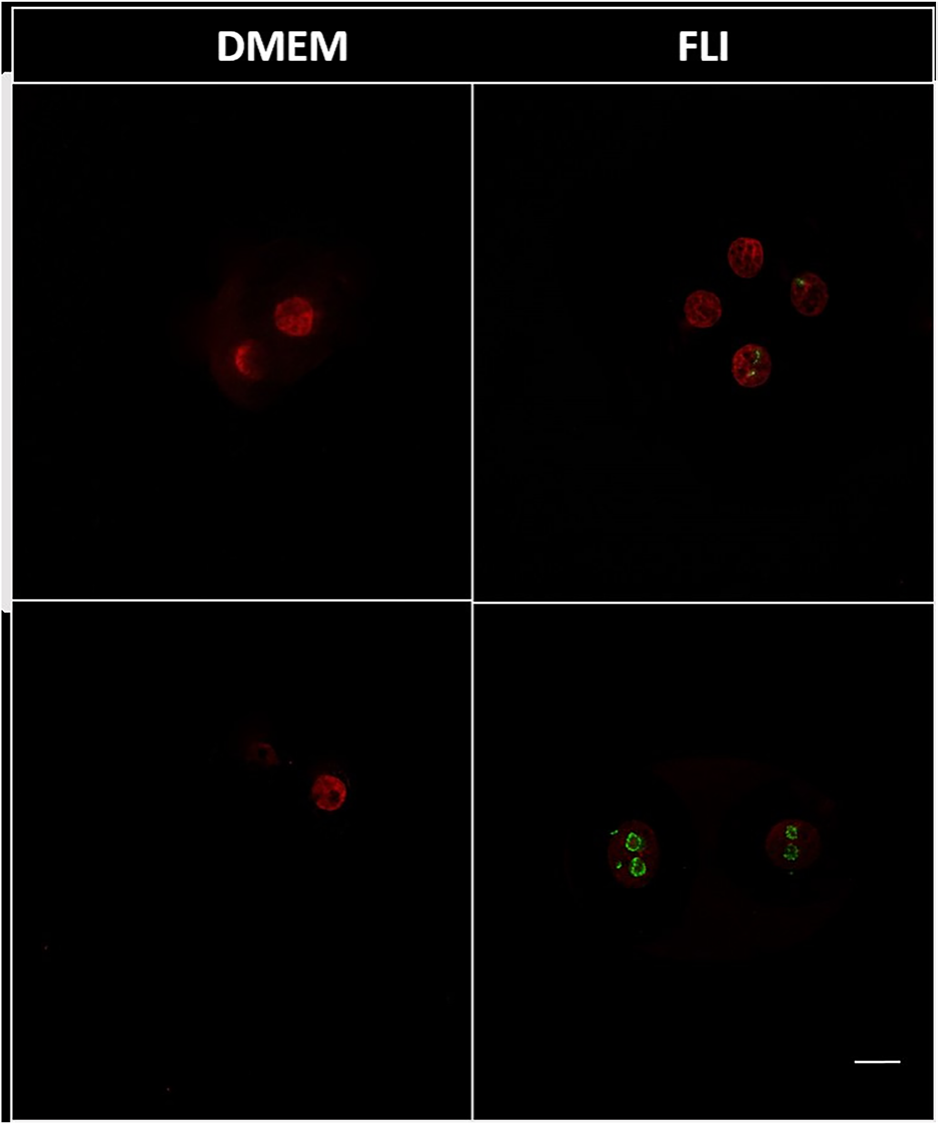
Positive UBF detection in 4 cell embryo from FLI experimental group. Scale bar represents 10 µm.

We detected UBF labelling in 4-cell embryos of the FLI experimental group, where positive labelling was visible in 72 inactive NPBs and 88 reticular nuclei observed in individual blastomeres (Fig. 3). We did not observe positive UBF labelling in 4-cell DMEM embryos. These results indicate activation of the embryonic genome in 4-cell embryos obtained by chemically activated oocytes from the FLI group.

### Analysis of global RNA synthesis and RNA polymerase I activity

**Figure 4:**
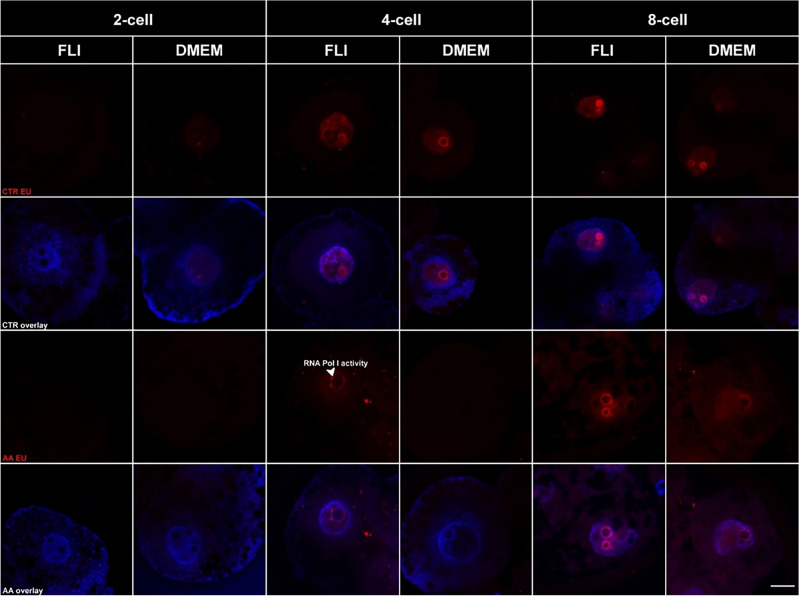
Global RNA synthesis and RNA polymerase I activity. *CTR* control groups, *AA* α-amanitine groups. The scale bar represents 10 µm.

The global RNA synthesis under the guidance of all RNA polymerases, especially RNA pol II corresponded to monitored embryo stages and EGA at the end of the 4-cell stage in both examined groups. However, in the groups where the α-amanitine was used, RNA Pol I activity was detected for the first time at the end of the 4-cell stage in the FLI group only. The situation in the 8-cell stage was similar and a clear signal was detected in the FLI group. On the other hand, the RNA polymerase I activity in the DMEM group was detected for the first time in 8-cell stages and this signal corresponds to the onset of RNA Pol I activity. These results indicate later nucleologenesis and slower onset of EGA in the DMEM group (Fig. 4).

### Transcriptomic profiles of embryos

We used next-generation RNA sequencing (RNA-Seq) for transcriptomic profiling of in vitro produced embryos. The statistical model contained data from the cell stage and maturation medium. Analysed embryos were used for comparison of the differences in the presence of transcripts between individual embryo stages. The reason for selecting the analysed 2-, 4- and 8-cell stages was that our primary hypothesis featured a positive influence of FLI maturation medium on the success rate of in vitro produced embryos.

**Figure 5:**
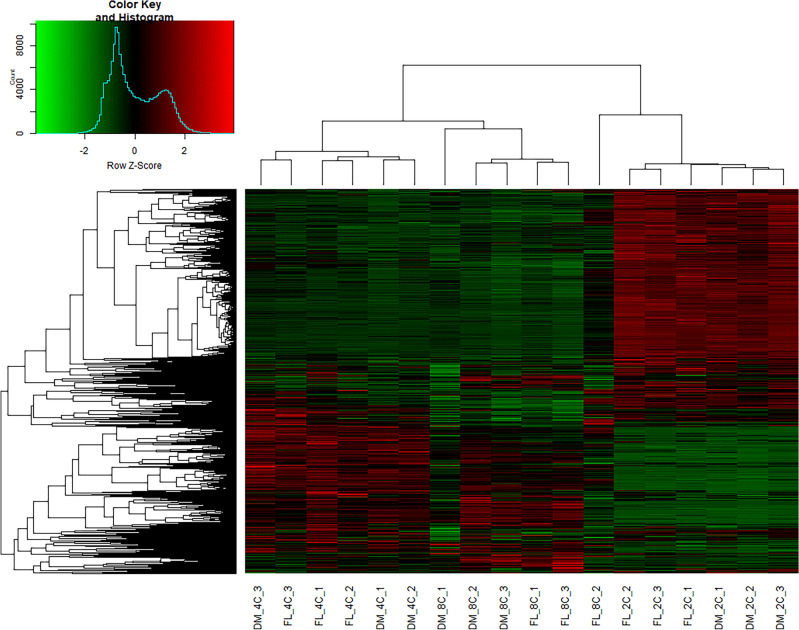
Analysis of grouping of differentially expressed genes of individual experimental groups.

To determine gene expression profiles related to developmental stages we have analysed RNA-Seq data of embryos using hierarchic grouping (Fig. 5). The presence of a gene in at least two out of three iterations for every experimental group was set as a gene detection standard.

We have decided to compare functional groups of genes and transcripts of interests between higher and lower stages of development and focus on statistically significant differences in the amount and function of genes (|Log2FoldChange| > 1.5 and p < 0.05). Gene lists from all experimental groups were analysed using DAVID.

Based on the immunocytochemical staining results, we analysed especially 4-cell FLI embryos. By comparing 4-cell FLI and DMEM embryos, we identified 6 different expressed genes, of which 3 genes were upregulated and 3 downregulated. Upregulated genes present in the 4-cell FLI embryo included the gene encoding lysine-specific demethylase 4E *(KDM4E*), the gene encoding centrosomal protein 161B (*FAM161B*), and the gene encoding *zona pellucida* glycoprotein 4 (*ZP4*). Downregulated genes in 4-cell FLI embryos included the gene encoding fatty acid binding protein 5 (*FABP5*), the gene encoding 40S ribosomal protein S27 (*RPS27*), and the gene encoding ubiquitin-conjugating enzyme 2W (*UBE2W*).

By analysing 4-cell FLI and DMEM embryos, we also identified the top 20 expressed genes (Table 3). All these genes and the proteins resulting from their translation are involved in mitochondrial OXPHOS and cell proliferation. Because of a similar pattern of UBF activity described in 8-cell DMEM embryos and 4-cell FLI embryos, we also compared differences between these stages.

**Table 3:** List of top 20 genes expressed in 4-cell embryos.

4-cell DMEM			4-cell FLI		
Gene ID	Gene name	TPM	Gene ID	Gene name	TPM
ENSSSCG00000038407	RF00002	222,432.5	ENSSSCG00000038407	RF00002	265,795.1
ENSSSCG00000034995	RF00017	59,730.0	ENSSSCG00000034995	RF00017	51,490.1
ENSSSCG00000018078	*COX2*	31,102.6	ENSSSCG00000018078	*COX2*	29,395.7
ENSSSCG00000018081	*ATP6*	18,068.6	ENSSSCG00000018081	*ATP6*	18,761.2
ENSSSCG00000019556	RF00100	15,960.4	ENSSSCG00000034113	novel gene	13,010.6
ENSSSCG00000018075	*COX1*	13,654.8	ENSSSCG00000018075	*COX1*	12,853.9
ENSSSCG00000034113	novel gene	12,733.8	ENSSSCG00000019556	RF00100	12,245.0
ENSSSCG00000018082	*COX3*	11,093.2	ENSSSCG00000018082	*COX3*	10,926.8
ENSSSCG00000018069	*ND2*	9807.1	ENSSSCG00000018069	*ND2*	8339.0
ENSSSCG00000018092	*ND6*	8977.3	ENSSSCG00000018065	*ND1*	6835.1
ENSSSCG00000018094	*CYTB*	8136.5	ENSSSCG00000018094	*CYTB*	6823.0
ENSSSCG00000018065	*ND1*	8021.4	ENSSSCG00000018087	*ND4*	6556.0
ENSSSCG00000018087	*ND4*	6583.2	ENSSSCG00000018084	*ND3*	5815.8
ENSSSCG00000018084	*ND3*	5191.8	ENSSSCG00000018092	*ND6*	5586.0
ENSSSCG00000018080	*ATP8*	5010.7	ENSSSCG00000018080	*ATP8*	5199.7
ENSSSCG00000018091	*ND5*	4996.3	ENSSSCG00000018091	*ND5*	4331.1
ENSSSCG00000016275	*NCL*	3423.7	ENSSSCG00000018086	*ND4L*	3735.3
ENSSSCG00000018086	*ND4L*	3362.7	ENSSSCG00000008433	*CALM2*	3222.5
ENSSSCG00000008433	*CALM2*	3338.3	ENSSSCG00000016275	*NCL*	3195.8
ENSSSCG00000032708	RF00017	3300.0	ENSSSCG00000040929	*TPT1*	2928.3

**Figure 6:**
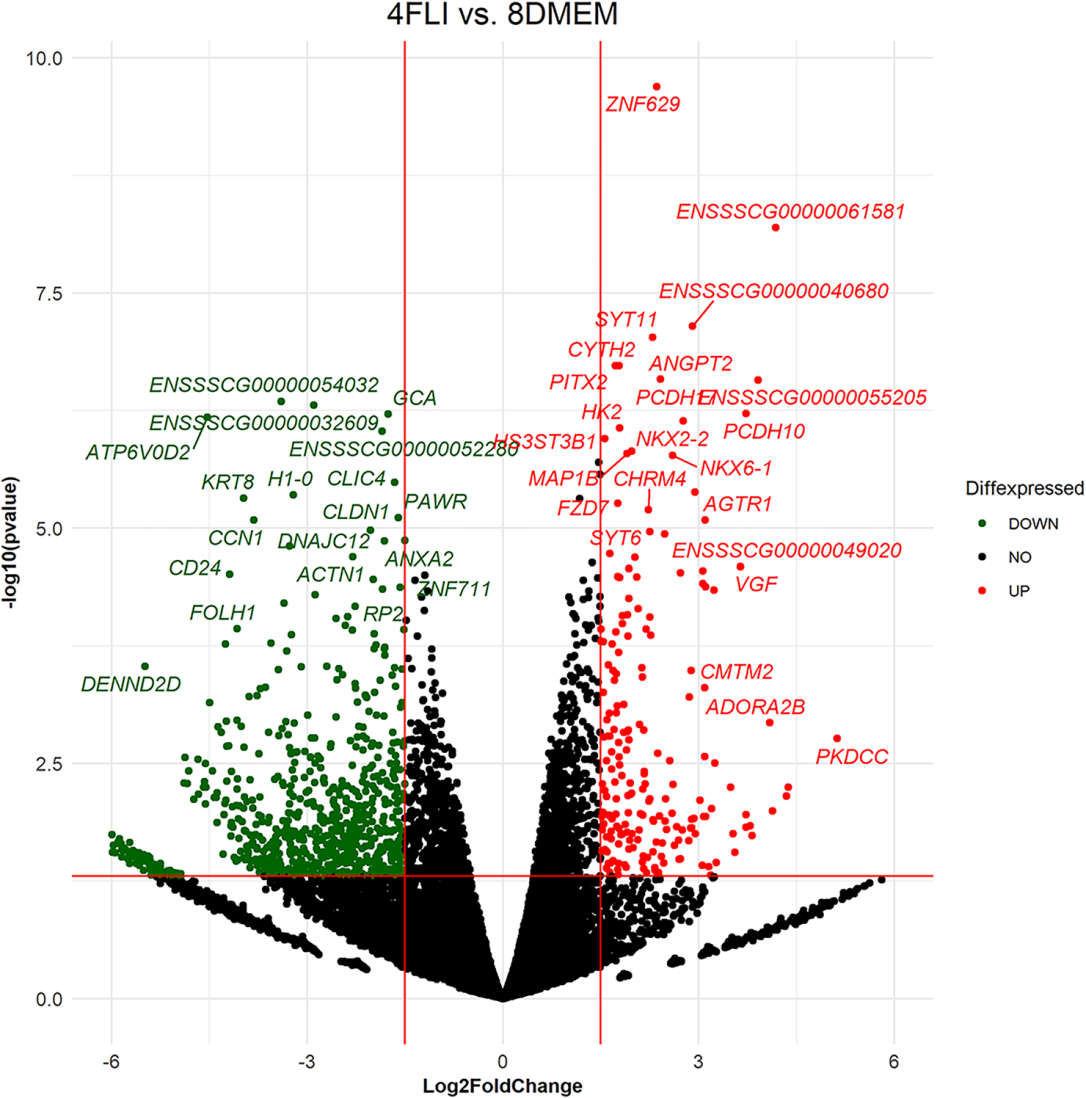
Volcano plot of detected genes between 4-FLI vs 8-DMEM embryos. Colourful points indicate the significantly different genes (Log2FoldChange| > 1.5 and p < 0.05).

By comparing 4-cell FLI embryos and 8-cell DMEM embryos, we detected 977 expressed genes, of which 204 genes were upregulated and 773 downregulated. The upregulated genes in 4-cell FLI embryos actively participated in essential biological processes centered primarily on cell proliferation, cell differentiation, and transcription regulation. A remarkable discovery is the negative regulation of DNA-template transcription, which supports our hypothesis of initiation of EGA during the 4-cell embryonic stage of parthenogenetically activated oocytes (Table 4, Fig. 6).

**Table 4:** List of biological pathways upregulated in 4-cell FLI in comparison to 8-cell DMEM embryos.

BP ID	BP term	Count	p-value
GO:0006357	Regulation of transcription from RNA polymerase II promoter	23	9.43E−06
GO:0000122	Negative regulation of transcription from RNA polymerase II promoter	10	8.37E−03
GO:0030154	Cell differentiation	9	2.89E−03
GO:0045892	Negative regulation of of transcription, DNA-templated	8	1.48E−03
GO:0008284	Positive regulation of cell proliferation	8	3.43E−03
GO:0043066	Negative regulation of apoptotic processes	7	8.62E−03
GO:00008285	Negative regulation of cell proliferation	5	7.34E−02
GO:0045596	Negative regulation cell differentiation	4	3.99E−04
GO:1900746	Positive regulation of p38mapk cascade	4	3.99E−04
GO:0045666	Positive regulation of neuron differentiation	4	4.91E−03

**Figure 7:**
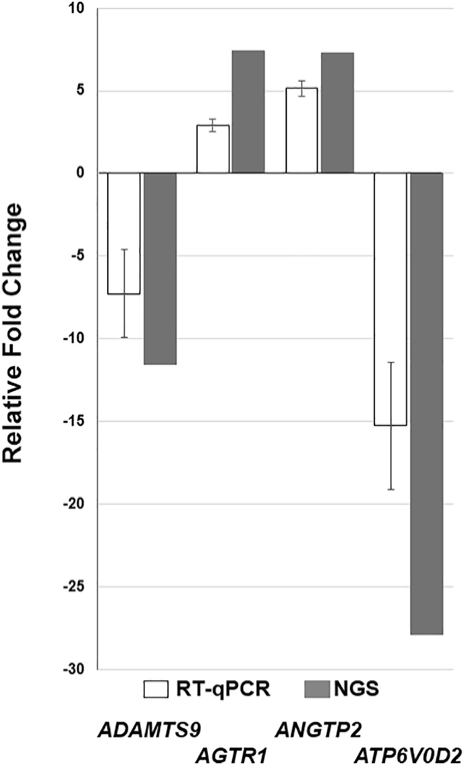
Gene expression of specific genes in experimental groups 4-FLI and 8-DMEM.

Sequencing validation was performed by RT-qPCR. During the validation process, we focused on investigating the gene expression of specific genes in experimental groups, 4-FLI and 8-DMEM. After analyzing the data, we established a coherent relationship between the RT-qPCR and NGS results, which are consistent with the results obtained (Fig. 7).

## Discussion

In general, the success rate of embryo production in vitro is lower than that of the in vivo environment. However, our laboratory has been working on improving this using porcine oocytes as a model system for research and testing methods that could be potentially applied in human ART. In a particular part of our study, we analysed the impact of IVM on selected oocyte and embryo parameters while comparing two cultivation media used for IVM—previously used DMEM medium and new FLI cultivation. Maturation rates of porcine oocytes in DMEM correlate with multiple outcomes achieved from different labs as Yuan, Krisher and Procházka et al. where the maturation success rate of up to 89.70% was attained in TCM-199. Maturation in FLI media results in a significantly higher level of MII oocytes^18,19^.

Parthenogenetic activation took place after the successful maturation. Oocytes were parthenogenetically activated by a combination of ionomycin and 6-DMAP. Our results are comparable to that of Prochazka et al*.* whose division results (81.4%) are close to our DMEM experimental group. Che et al.^20^ reported a similar outcome (71% cleavage vs. 32% blastocysts). In comparison to the FLI group, we achieved significantly higher rates in all parameters. We also determined different timings during early embryogenesis in experimental groups. Embryo division was faster in the FLI group and was similar to in vivo timing^21^. A similar positive effect of FLI medium in the oocyte maturation was also observed by Procházka et al., Serano Albal et al*.*, Currin et al*.*^6,19,22^. The quality of blastocyst-stage embryos was also enhanced and the efficiency of cattle and sheep in vitro production (IVP) as well as embryo quality were improved through FLI supplementation^23,24^.

Meiotic maturation in mammalian oocytes is a complex process that involves transcription silencing, germinal vesicle breakdown (GVBD) followed by extensive rearrangement of microtubules and actin filaments as well as other cytoskeleton-associated proteins providing the framework for various dynamic processes^25^. Also, it is expected that the important information necessary for full meiotic and developmental competence of oocytes be retained at the level of RNAs (maternal transcripts) and proteins. Based on this information we decided to apply the proteomic approach to identify groups of proteins that are differentially expressed in porcine oocytes maturated in qualitatively different maturation media (DMEM vs. FLI). The most significant differences in the number of identified proteins between experimental groups of oocytes were observed in the group of proteins (GE) involved in gene expression processes (24 in the DMEM and 8 in the FLI group). The loss and degradation of some GE proteins in the late phase of oocyte maturation can be connected with active silencing of transcription, however part of these proteins remains as a maternal deposit for further fertilisation, post-fertilisation, and early embryonic processes, especially embryonic genome activation. This group of proteins predominantly consists of proteins related to translational processes, mRNA processing, and oocyte-specific RNA binding proteins^26^.

The second group of proteins with slightly different numbers in DMEM and FLI oocytes consisted of cytoskeletal proteins (part of microtubules and microfilaments). It is already known that after GVBD, microtubules are condensed around the chromosomes and begin to migrate to the cortex, while microfilaments are densely accumulated in the subcortical region of oocytes, especially around the meiotic spindle of the oocyte^27,28^. In MII-stage mature mouse oocytes, microtubules and microfilaments mainly accumulate in the cortical cytoplasmic region. Microfilament and microtubular networks play a crucial role in the control of the asymmetric division of the oocyte during meiosis and thus ensure the accurate segregation of the maternal genome and asymmetric partitioning of cytoplasm with maternal stores necessary for embryonic development^29,30^. Therefore, the deposit of cytoskeletal proteins during the last phases of oocyte maturation should be more stable compared to the group of proteins involved in gene expression.

After observing the differences in first-division timing, we tried to detect the activation of an embryonic genome via immunocytochemistry. We focused on the marking of upstream binding factor transcription factor (UBF), which is essential for rRNA gene transcription. In the case of in vivo, the embryonic genome in pigs is activated during the third cell cycle (e.g. 4-blastomere stage), whereas in in vitro it happens at the 8-blastomere stage^31^. We observed positive marking in the 8-cell DMEM embryos, whereas in the second experimental group (FLI) the UBF marking was observable in 4-cell embryos. This conclusion displays the activity of the embryonic genome of 4-blastomere embryos obtained by parthenogenetic activation of oocytes maturated in FLI medium.

Based on the previous results supporting our hypothesis that the composition of FLI medium significantly contributes to more rapid activation of the embryonic genome and eliminates differences between in vitro and in vivo conditions, we realised further analysis at transcriptome level. We decided to use the new generation RNA sequencing (RNA-Seq) to determine the transcriptomic profiles of individual experimental groups, which helped us to detect the most important signalling pathways that were activated in the selected groups. Understanding signalling pathways involved in early embryogenesis can provide us with information about the basic mechanisms of cell functioning and interactions necessary for morphogenesis and organogenesis. In general, some expression patterns were similar to previous studies^32,33^. By subsequent comparison of individual stages, we reached multiple conclusions. Comparing the 4-cell FLI to the 4-cell DMEM, we observed different expression of 6 genes. Upregulated genes especially the *KDM4E* gene belong to the family of lysine-specific demethylases, which take part in the catalytic function and handle chromatin remodelling. *FAM161B* belongs to the FAM family of genes responsible for the development of retinal progenitors during embryogenesis. *ZP4* is the fourth component of the *zona pellucida.* The difference in ZP4 transcript abundance suggests that the structural properties provided by this protein are required to ensure proper protection of embryos during development^34^. The *FABP5* protein belongs to a family of fatty acid-binding proteins and may play a role in the absorption, transport, and metabolism of fatty acids. *RPS27* is a structural component of ribosomes and takes part in the biogenesis of the small ribosomal subunit. Its overexpression leads to spontaneous apoptosis and can inhibit cell growth^35^. *UBE2W* mediates N-terminal ubiquitination, which is very important in protein quality control, cell cycle control, proliferation, development, signal transduction, and DNA repair^36^. The top 20 common genes between 4-cell FLI and DMEM are involved in mitochondrial OXPHOS and cell proliferation, which can be concluded that FLI embryos have higher energy requirements due to their similarities with the 8-cell stage^37–39^.

To verify the progress of 4-cell FLI embryos, we decided to compare this group with 8-cell DMEM embryos. In the individual experimental groups, we recorded at 4-cell stage genes involved in cell proliferation, differentiation, and negative DNA-template transcription. This fact suggests that maternal factors are replaced by their embryonic production at this stage, which reflects the number of transcripts related to mitochondrial activity, metabolism cell proliferation, and differentiation. We assume that these important differences are related to the influence of the media composition during IVM of oocytes. Changes in the oocyte quality support early embryogenesis, which is significantly successful and more progressive, which correlates with our results.

## Conclusion

Based on our results, it is possible to evaluate the importance of the maturation medium in IVP embryos. In this work, we demonstrated an undeniably positive influence of the FLI medium by achieving significantly different results in all analysed experiments. We observed the positive influence of FLI maturation medium in significantly different results within IVM oocytes, division, and proportion of blastocysts. By analysing the resulting embryos, we confirmed the influence of the maturation medium on the level of UBF and RNA pol I, II activity, a proteomic profile supporting the progressive nature of embryos formed from oocytes matured in the FLI medium. In addition, by determining the transcriptome profiles, we confirmed the progressivity of the embryos by confirming the EGA in the 4-cell embryo by detecting the embryonic production manifested by the number of transcripts, protein metabolism and their transport. These results show that the use of a chemically defined FLI maturation medium brought the efficiency of in vitro embryo production closer to that achieved in an in vivo environment.

## Methods

Except otherwise specified, all chemicals, culture media, and supplements were obtained from Sigma Chemical Co. (Munich, Germany). At least three repetitions were analysed in each experiment, except otherwise stated. Nunc provided all the plastic materials (Roskilde, Denmark). Before use, the media were freshly produced, pre-warmed at 38.5 °C, and filtered with a 0.22 m Whatman membrane filter. Our research was based on the differences between two maturation media. The comparison between them was examined by evaluation of the ability to reach the metaphase of the second meiotic division (MII phase), cleavage rate, blastocyst rate, proteomic profiling of oocytes, immunodetection of embryonic genome activation (EGA), cytoplasmic structures detection by transmission electron microscopy (TEM) and RNA-Seq analysis.

### Cumulus-oocytes collection

The ovaries were obtained from a local slaughterhouse, specifically from premature gilts. The ovaries were removed, and brought to the lab in a thermo-flask at 38 °C. Only medium-sized antral follicles with a diameter of 3–6 mm were aspirated by a syringe attached to a 20 G needle. Follicular fluid was collected in a test tube and left to settle for 10 min. The cumulus-oocyte complexes (COCs) that were surrounded by a dense multi-layered cumulus were collected and rinsed twice with PXM-Hepes (HEPES buffered porcine X medium)^19,40,41^.

### In vitro maturation

After collection, the COCs were washed thrice in maturation media and cultivated in 500 µl of media. The first medium was standard Dulbecco’s modified Eagle’s medium (DMEM, Biowest, L0101-500) supplemented with 10 IU/ml PMSG (Prospec, Israel) and hCG (Prospec, Israel), 50 ng/ml EGF, 100 ng/ml IGF1 and 5 ng/ml FGF. The second medium was upgraded FLI medium composed of TCM 199, 22 ng/ml Na-pyruvate, 6,85 mM l-glutamine, 0.57 m ML-cysteine, 50 µg/ml Gentamycin, 1 mg/ml BSA, 10 IU/ml EGF, 40 ng/ml FGF2, 20 ng/ml IGF1, 20 ng/ml LIF, 10 IU/ml PMSG and hCG (Prospec, Israel) for 44 h at 38.5 °C with 5% CO_2_^17,42^.

### Evaluation of MII stage

After 44 h of IVM, gentle pipetting was used to remove cumulus cells (CCs) from oocytes to determine the phase of their nuclear maturation. The denuded oocytes were put on slides and preserved in an ethanol-acetic acid solution (3:1) for 48 h. After that, the oocytes were dyed with 1% lacmoid and examined under a light microscope^43^.

### Sample preparation for proteomic profiling

We used samples of 20 mature oocytes from DMEM and FLI media for nano HPLC-Chip-MS/MS analysis. Oocytes in each group were lysed by shaking for 1 h at 37 °C in 200 μl of SDT lysis buffer containing 4% SDS (w/v), 0.1 M Tris/HCl pH 7.6, 0.1 M DTT and protease inhibitors (1 × Roche complete). Immediately after lysis, samples were cooled down to 4 °C and centrifuged at 16,000×*g* for 5 min The obtained supernatant was transferred to filter plates with a cut-off molecular weight of 3 kDa (Amicon Ultra-0.5 ml 3 K, Millipore) and centrifuged at 14,000×*g* for 40 min. A modified filter-assisted sample preparation protocol (FASP) was used to extract tryptic peptides from a given complex protein mixture^44^. Detergent was removed by buffer exchange in two consecutive washes of the filter plates with 200 μl of 8 M urea in 0.1 M Tris/HCl pH 8.5 (UA), each followed by centrifugation at 14,000×*g* for 40 min Flow-through was removed from the collection tubes. Proteins were subsequently alkylated in 100 µl of 0.05 M iodoacetamide in UA by mixing at 600 rpm in a thermo-mixer for 1 min and incubating without mixing for 20 min After centrifugation at 14,000×*g* for 30 min, two additional washes using 100 µl of 0.05 M NH_4_HCO_3_, with 10 min at 14,000×*g* after each wash, were included to remove excess urea and prepare the proteins for digestion. Protein digestion was performed by adding 2 µg of trypsin (Trypsin Gold, Promega) in 0.05 M NH_4_HCO_3_ and incubating at 37 °C overnight. Peptides were recovered by spinning the filter plates upside down at 14,000×*g* for 40 min The mixture was dried under vacuum and the pellet was resuspended in 50 µl of mobile phase (97% water, 3% acetonitrile)^11^.

### Protein identification by tandem mass spectrometry (nano HPLC-chip-MS/MS)

A 40 nL enrichment column was used to load tryptic peptides (loaded with Zorbax SB C18, 5 μm) to an Agilent 1260 ChipCube MS interface using an Agilent 1260 capillary pump (Agilent Technologies, Palo Alto, USA). After loading and desalting the sample in the chip enrichment column, the peptides were eluted by direct flushing and transferred to the analytical column at a flow rate of 600 nL/min by an Agilent 1260 Nano pump with increasing percentages of the organic phase. The mobile phase used for chromatography included an aqueous solution of formic acid (0.1%, v/v) or acetonitrile. The separation process used a gradient elution method according to the following sequence: 0 min with 3% B; 2 min with 3% B; 25 min with 50% B; 30 min with 50% B; 35 min with 95% B; 40 min, 95% B; 45 min, 3% B, followed by a ten-minute column re-equilibration period in the end. The analytical column was connected to an Agilent 6500 Series Q-TOF mass spectrometer. A voltage of 1750 V was applied to the electrodes of the nanospray ionization chamber. Highly pure nitrogen (99.99%) was used as the collision gas, and the collision energy was fixed as a function of the mass and charge of the precursor ion. MS/MS spectra were obtained by automatically switching between MS and MS/MS mode (auto MS/MS mode). The obtained MS/MS data were analyzed using the SpectrumMill search engine (Agilent Technologies, Palo Alto, USA). The hand-built UniProt porcine proteome database was used for the search. (UP000008227). SpectrumMill software used specific automatic validation criteria to validate the identified proteins and peptides. These included a minimum protein score of 10 and minimum scores for spectra produced by fragmentation of precursor ions with 2 +, 3 +, and 4 + charge states. For the spectral score, the required values were at least 8, 7, and 9. In addition, a maximum intensity value of at least 60% was required^11^.

### Parthenogenetic activation

As a consequence of problems with polyspermy, porcine parthenogenetic embryos are presently used instead of in vitro fertilisation (IVF) for embryological research. For that, CCs were mechanically removed using a 100 µl pipette and oocytes were washed twice in PXM-Hepes. Oocytes were stimulated by being exposed to 10 µM ionomycin in PXM-Hepes for 5 min After being washed twice in porcine zygote medium 3 (PZM-3) supplemented with 2 mM 6-dimethylaminopurine (6-DMAP), they were cultivated for 5 h at 38.5 °C with 5% CO_2_ in the atmosphere. A total of 50 potential parthenotes were cultivated for 144 h in 4-well dishes after being washed twice in 500 µl PZM-3^41^.

### Immunocytochemistry

Experimental groups were composed of 2-,4- and 8-blastomere embryos obtained by chemical parthenogenetic activation of oocytes maturated in DMEM and FLI maturation medium. We have determined the presence of upstream binding factor (UBF, Abnova H00007343-MO1, TW) using immunocytochemistry. We used Tyrode's solution in PBS containing 2% BSA (Merck, 1120180100, Germany) to permeabilize the zona pellucida, which improves the accessibility and staining efficiency of antibodies, allowing for more accurate visualization of specific proteins. Subsequently, the embryos were fixed for 40 min at room temperature using a 4% paraformaldehyde solution. After fixation, we performed three washes with PBS containing 2% BSA. To permeabilize the samples for 90 min, we utilized a solution of 0.5% Triton X-100 in PBS containing 2% BSA (AppliChem Panreac, A09649050). Following blocking, we exposed the samples overnight at 4 °C to a specific primary antibody (Abnova, UBFT H00007343-M01), which was diluted in PBS containing 2% BSA at a ratio of 1:50. After exposure, we washed the samples for thirty minutes with PBS containing 2% BSA at room temperature and then incubated them in darkness for two hours at room temperature with a blocking solution that included Alexa Fluor594 anti-rabbit secondary antibody (1:350). After performing three additional washes, we transferred the samples onto glass slides and covered them with glass coverslips using Vectashield mounting medium with DAPI (Vectashield hardest antifade mounting medium with DAPI; H-1500) solution. Finally, the samples were evaluated using a CarlZeiss LSM710 confocal microscope.

### Analysis of global RNA synthesis and RNA polymerase I activity

The embryos were processed by Click-iT RNA Imaging Kit (Invitrogen, UK) and optimized protocol. Briefly, for analysis of global RNA synthesis embryos were cultivated in a PZM-3 medium supplemented with 1 mM 5-ethynyl uridine (EU) for 2.5 h before the fixation. The embryos were fixed in 4% Paraformaldehyde at the end of the 2-, 4-, and 8-cell stages. 2-cell stage embryos were fixed 30 h after the parthenogenetic activation (PA), 4-cell stage embryos were fixed 66.5 h after the PA and 8-cell stage embryos were fixed 77 h after the PA. Standard EGA timing and porcine embryo division were taken into account for selecting the timing of the fixation^45,46^. The timing of porcine embryo development was analysed by CytoSMART Lux2 (CytoSMART Technologies, NL). For analysis of RNA polymerase I activity, the timing of the EU staining protocol and fixation remained unchanged, but PZM-3 medium was supplemented with 25 μg/ml α-amanitin 6 h before the fixation. Samples were mounted in ProLong Gold Antifade Mountant with DAPI (ThermoFisher Scientific, Slovakia). The evaluation was processed under the confocal laser scanning microscope Zeiss LSM 710 (Zeiss, Germany), and the images were captured by ZEN Black software (Zeiss, Germany) and processed by ImageJ software and Zoner Photo Studio 18.

### Samples preparation, RNA isolation, and library preparation for sequencing

For analyses associated with mRNA extraction, which is essential for ascertaining gene expression, it was necessary to collect embryos at 2-cell, 4-cell, and 8-cell stages. Pooled embryos in three replicates from each stage (20 pcs) were rinsed twice in PBS + 2% BSA, and in pure PBS, lysed in 100 µl of lysis solution, and stored at -80 °C.

Total RNA was extracted by using an RNAqueous Micro kit (Thermo Fisher, AM1931) according to the manufacturer’s instructions. RNA quality control was done using Bioanalyzer 2100 with the Bioanalyzer RNA 6000 pico assay kit. The amount of RNA was measured by a Qubit 2.0 Fluorometer with Qubit™ RNA High Sensitivity kit. SMARTer Stranded Total RNA-Seq Kit v2-Pico Input Mammalian Kit (Takara, France) was used for RNA library construction. Libraries quality and quantity were assessed using Bioanalyzer 2100 with a High Sensitivity DNA assays kit and a Qubit dsDNA High Sensitivity kit. Libraries were sequenced on NovaSeq 6000 according to the manufacturer’s protocol using NovaSeq 6000 SP Reagent kit (200 cycles) using a mode for reading at the end of pairs with a length of reading 2 × 100 base pairs (bp). Collected biological data were demultiplexed and FASTQ files were generated for each sample using the FastQC tool. Individual sequences were mapped and annotated to the reference swine genome (*Sus scrofa*) using bowtie2. Consequently, we have calculated expression values for each gene using the Illumina Dragen software. Differential analysis of expression was done using the statistic software package DESeq2. Heatmap and volcano plot were generated using the R package. The bioinformatics web tool Database for Visual and Integrated Discovery (DAVID) was used for detecting biological processes in which differentially expressed genes were involved.

### RT-qPCR expression analysis of embryo genes

To validate the sequencing data, the samples of embryos were collected and RNA was isolated as indicated in the paragraph above. A selection of potential target genes was made based on the sequencing outcomes. Primers for these chosen genes were created using Beacon Designer v. 8.21 software and are detailed in Table 5. One-step RT-qPCR analysis was conducted on a RotorGene 3000 cycler (Corbett Research) employing the QIAGEN OneStep RT-PCR Kit (Qiagen) within a 20 µl reaction mix comprising specified components including RNase-free water. The thermal cycling process encompassed stages such as reverse transcription at 50 °C for half an hour, initial denaturation at 95 °C for a quarter of an hour; subsequent PCR cycles involving denaturation at 94 °C for fifteen seconds, annealing at primer-specific temperatures for fifteen seconds and extension at 72 °C for twenty seconds; concluding with a final extension step of five minutes at 72 °C. Fluorescence readings were captured after each extension phase to track product amplification progress while verification involved melting analysis and electrophoresis on agarose gel utilizing MidoriGreen Direct (Nippon Genetics).

**Table 5:** List of primers used for RT-qPCR.

Gene transcript	Primers 5′–3′	Amplicon length (bp)	T_an_ (°C)	GenBank accession number
*ADAMTS9*	GGA TGA TAA CTA CTT AGG T TAC TCT ATT ACG GCA TTC	160	55	XM_013981851
*AGTR1*	TTG GTG GTA ATT GTC ATT ATT CCA TAG CAG TGT AGA	133	56	XM_003132469
*ANGPT2*	AGG CTT ACT CAC TGT ATG TTG GCT TAT GCT GCT TAT	110	56	NM_213808
*ATP6V0D*	CAT TAT TGA CAC AGA GAT CAT CAG TAG AAT CAC ATT	226	55	XM_003125581
*H2AZ1*	GGC CGT ATT CAT CGA CAC CTG ACC AGC GAT TGT AGC CTT GAT	231	56	NM_001123122
*YWHAG*	CAG CCC ACT CAC CCC ATT AG TGC TGA TCG CTT GTC CAG AG	218	56	XM_005661962

*T*_*an*_ annealing temperature.

### Statistic evaluation

The analysis of oocyte maturation and embryo development involved conducting a one-way ANOVA (Anova single factor) or t-test ((SigmaPlot 12.0, London, UK) with Tukey’s post-test, depending on the number of groups. Comparisons of transcript levels were made using a t-test. The standard error of the mean (SEM) is shown as error bars. Probability values below 0.05 were deemed to be statistically significant.

## Data Availability

The datasets generated and analyzed during the current study are available in the PRIDE repository (PXD045481) and NCBI's Gene Expression Omnibus^47^ and are accessible through GEO Series accession number GSE244075.

## References

[1] Fowler KE, Mandawala AA, Griffin DK, Walling GA, Harvey SC (2018). The production of pig preimplantation embryos in vitro: Current progress and future prospects. Reprod. Biol..

[2] Hryhorowicz M (2020). Application of genetically engineered pigs in biomedical research. Genes.

[3] Prather RS (2007). Targeted genetic modification: Xenotransplantation and beyond. Clon. Stem Cells.

[4] Dujíčková L, Makarevich AV, Olexiková L, Kubovičová E, Strejček F (2021). Methodological approaches for vitrification of bovine oocytes. Zygote.

[5] De Roo C, Tilleman K (2021). In vitro maturation of oocytes retrieved from ovarian tissue: Outcomes from current approaches and future perspectives. J. Clin. Med..

[6] Currin L (2022). Optimizing swine in vitro embryo production with growth factor and antioxidant supplementation during oocyte maturation. Theriogenology.

[7] Kinterova V, Kanka J, Petruskova V, Toralova T (2019). Inhibition of Skp1-Cullin-F-box complexes during bovine oocyte maturation and preimplantation development leads to delayed development of embryos. Biol. Reprod..

[8] Murin M (2023). Porcine oocytes matured in a chemically defined medium are transcriptionally active. Theriogenology.

[9] Chen PR, Redel BK, Kerns KC, Spate LD, Prather RS (2021). Challenges and considerations during in vitro production of porcine embryos. Cells.

[10] van der Weijden VA (2021). Transcriptome dynamics in early in vivo developing and in vitro produced porcine embryos. BMC Genom..

[11] Bartkova A (2020). Characterization of porcine oocytes stained with Lissamine Green B and their developmental potential in vitro. Anim. Reprod..

[12] Motta L, Chaves D, Bhat M (2018). In vitro embryo production in the pig. Reprod. Biotechnol. Farm Anim..

[13] Edwards RG, Bavister BD, Steptoe PC (1969). Early stages of fertilization in vitro of human oocytes matured in vitro. Nature.

[14] Hatırnaz Ş (2018). Oocyte in vitro maturation: A sytematic review. TJOD.

[15] Vuong LN (2020). Live births after oocyte in vitro maturation with a prematuration step in women with polycystic ovary syndrome. J. Assist. Reprod. Genet..

[16] Nemcova L (2023). Importance of Supplementation during In Vitro Production of Livestock Animals.

[17] Yuan Y (2017). Quadrupling efficiency in production of genetically modified pigs through improved oocyte maturation. Proc. Natl. Acad. Sci. U SA.

[18] Yuan Y, Krisher RL (2012). In vitro maturation (IVM) of porcine oocytes. Methods Mol. Biol..

[19] Procházka R (2021). The role of MAPK3/1 and AKT in the acquisition of high meiotic and developmental competence of porcine oocytes cultured in vitro in FLI medium. IJMS.

[20] Che L, Lalonde A, Bordignon V (2007). Chemical activation of parthenogenetic and nuclear transfer porcine oocytes using ionomycin and strontium chloride. Theriogenology.

[21] Hyttel P (2000). Nucleolar proteins and ultrastructure in preimplantation porcine embryos developed in vivo. Biol. Reprod..

[22] Serrano Albal M (2022). Supplementation of porcine in vitro maturation medium with FGF2, LIF, and IGF1 enhances cytoplasmic maturation in prepubertal gilts oocytes and improves embryo quality. Zygote.

[23] Stoecklein KS, Ortega MS, Spate LD, Murphy CN, Prather RS (2021). Improved cryopreservation of in vitro produced bovine embryos using FGF2, LIF, and IGF1. PLoS ONE.

[24] Tian H (2021). Enhancing the developmental competence of prepubertal lamb oocytes by supplementing the in vitro maturation medium with sericin and the fibroblast growth factor 2-leukemia inhibitory factor: Insulin-like growth factor 1 combination. Theriogenology.

[25] Roth Z, Hansen PJ (2005). Disruption of nuclear maturation and rearrangement of cytoskeletal elements in bovine oocytes exposed to heat shock during maturation. Reproduction.

[26] Gegenfurtner K, Flenkenthaler F, Fröhlich T, Wolf E, Arnold GJ (2020). The impact of transcription inhibition during in vitro maturation on the proteome of bovine oocytes†. Biol. Reprod..

[27] Verlhac M-H, Terret M-E (2016). Oocyte maturation and development. F1000 Res..

[28] Terada Y (2005). Cytoskeletal dynamics during mammalian gametegenesis and fertilization: Implications for human reproduction. Reprod. Med. Biol..

[29] Gumus E, Bulut HE, Kaloglu C (2010). Cytoskeletal changes in oocytes and early embryos during in vitro fertilization process in mice. Anat. Histol. Embryol..

[30] Mao L, Lou H, Lou Y, Wang N, Jin F (2014). Behaviour of cytoplasmic organelles and cytoskeleton during oocyte maturation. Reprod. Biomed. Online.

[31] Østrup O (2013). RNA profiles of porcine embryos during genome activation reveal complex metabolic switch sensitive to in vitro conditions. PLoS ONE.

[32] Zhang C (2021). The dynamic changes of transcription factors during the development processes of human biparental and uniparental embryos. Front. Cell Dev. Biol..

[33] Niakan KK, Eggan K (2013). Analysis of human embryos from zygote to blastocyst reveals distinct gene expression patterns relative to the mouse. Dev. Biol..

[34] Lamas-Toranzo I (2019). ZP4 confers structural properties to the zona pellucida essential for embryo development. Elife.

[35] Wang R (2015). Loss of function mutations in RPL27 and RPS27 identified by whole-exome sequencing in Diamond-Blackfan anaemia. Br. J. Haematol..

[36] Qi C (2015). Biochemical and structural characterization of a novel ubiquitin-conjugating enzyme E2 from *Agrocybe aegeria* reveals Ube2w family-specific properties. Sci. Rep..

[37] May-Panloup P, Boguenet M, El Hachem H, Bouet P-E, Reynier P (2021). Embryo and its mitochondria. Antioxidants.

[38] Babayev E, Seli E (2015). Oocyte mitochondrial function and reproduction. Curr. Opin. Obstet. Gynecol..

[39] Mao X (2019). Genetic diversities of MT-ND3 and MT-ND4L genes are associated with high-altitude adaptation. Mitochondrial. DNA B.

[40] Ireland JJ, Murphee RL, Coulson PB (1980). Accuracy of predicting stages of bovine estrous cycle by gross appearance of the corpus luteum. J. Dairy Sci..

[41] Yoshioka K, Suzuki C, Tanaka A, Anas IM-K, Iwamura S (2002). Birth of piglets derived from porcine zygotes cultured in a chemically defined medium. Biol. Reprod..

[42] Lucas-Hahn A (2018). 122 a new maturation medium improves porcine embryo production in vitro. Reprod. Fertil. Dev..

[43] Laurincik J, Rath D, Niemann H (1994). Differences in pronucleus formation and first cleavage following in vitro fertilization between pig oocytes matured in vivo and in vitro. J. Reprod. Fertil..

[44] Wiśniewski JR, Zougman A, Nagaraj N, Mann M (2009). Universal sample preparation method for proteome analysis. Nat. Methods.

[45] Arrell VL, Day BN, Prather RS (1991). The transition from maternal to zygotic control of development occurs during the 4-cell stage in the domestic pig, *Sus Scrofa*: Quantitative and qualitative aspects of protein synthesis1. Biol. Reprod..

[46] Cao S (2014). Specific gene-regulation networks during the pre-implantation development of the pig embryo as revealed by deep sequencing. BMC Genom..

[47] Edgar R, Domrachev M, Lash AE (2002). Gene expression omnibus: NCBI gene expression and hybridization array data repository. Nucleic Acids Res..

